# Interlocking design, programmable laser manufacturing and testing for architectured ceramics

**DOI:** 10.1038/s41598-022-22250-9

**Published:** 2022-10-15

**Authors:** H. Yazdani Sarvestani, I. Esmail, Z. Katz, S. Jain, J. H. Sa, D. Backman, B. Ashrafi

**Affiliations:** 1 Aerospace Manufacturing Technology Center, National Research Council Canada, 5145 Decelles Avenue, Montreal, QC H3T 2B2 Canada; 2 Structures and Materials Performance Laboratory, National Research Council Canada, 1200 Montreal Road, Ottawa, ON K1A 0R6 Canada

**Keywords:** Aerospace engineering, Mechanical engineering

## Abstract

Tough and impact-resistant ceramic systems offer a wide range of remarkable opportunities beyond those offered by the conventional brittle ceramics. However, despite their promise, the availability of traditional manufacturing technique for fabricating such advanced ceramic structures in a highly controllable and scalable manner poses a significant manufacturing bottleneck. In this study, a precise and programmable laser manufacturing system was used to manufacture topologically interlocking ceramics. This manufacturing strategy offers feasible mechanisms for a precise material architecture and quantitative process control, particularly when scalability is considered. An optimized material removal method that approaches near-net shaping was employed to fabricate topologically interlocking ceramic systems (load-carrying assemblies of building blocks interacting by contact and friction) with different architectures (i.e., interlocking angles and building block sizes) subjected to low-velocity impact conditions. These impacts were evaluated using 3D digital image correlation. The optimal interlocked ceramics exhibited a higher deformation (up to 310%) than the other interlocked ones advantageous for flexible protections. Their performance was tuned by controlling the interlocking angle and block size, adjusting the frictional sliding, and minimizing damage to the building blocks. In addition, the developed subtractive manufacturing technique leads to the fabrication of tough, impact-resistant, damage-tolerant ceramic systems with excellent versatility and scalability.

## Introduction

Tough and impact resistant ceramic systems represent an ongoing revolution in materials and structures for aerospace, marine, automotive, construction, and armour applications^1^. Their outstanding properties (e.g. low-density, high compressive strength, high thermal stability, and high oxidation and corrosion resistance) as well as enhanced toughness and multi-impact resistance offer unique advantages over conventional rigid ceramic systems. Among numerous possibilities, tough ceramics have emerged as an ideal candidate for the extreme thermo-mechanical conditions such as thermal protection systems in gas-turbine engines, leading edge, or nozzle engine components^2^. Architectured ceramics, in particular, have attracted significant attention owed to their high mechanical performance (i.e., stiff, tough, multi-impact resistant, and damage tolerant) in advanced engineering applications^3^. The intrinsic rigid and brittle nature at the individual component level can be successfully translated to the enhanced toughness at the overall structural level via bioinspiration, as seen in biological materials such as bone^4^, nacre^5^, tooth enamel^6^, or sponge spicules^7^. Of the bioinspiration strategies which offer a toughness enhancement, the “topologically interlocking concept” consists of hard and stiff building blocks bonded along weak interfaces^8–10^. The challenge lies in the precise and industrially-scalable manufacturing of such mechanically enhanced structures^8,11–13^.

Both advanced subtractive and additive manufacturing technologies have emerged as promising solutions for the fabrication of the architectured ceramics with sophisticated architectured designs^14,15^. The subtractive manufacturing technique considered encompasses the use of advanced laser systems to develop three-dimensional (3D) architectures in brittle materials (e.g. glass), resulting in the improved resistance against quasi-static and low-velocity impact loads^16^. However, there are considerable drawbacks to the use of subtractive manufacturing technologies for the machining of brittle materials. Examples of these engineering hurdles include the complexity of process parameter optimization for various and differing laser setups, material compositions and thicknesses, and geometric/topological objectives. There has been significant investigation into the parametric effects of various fiber laser parameters, including the effect of fluence on ablation rate^17,18^, of raster pitch on surface roughness^19^, and of traverse speed and focal position on cut quality^20^. Findings from this research have led to the minimization of ripples and the elimination of cracks during ablation cutting. Although many studies have been performed on the design and assembly (e.g. machining, casting, or additive manufacturing) of topologically interlocked glasses or ceramics^10,21^, less consideration has been given to developing precise, near-net shaping and industrially scalable subtractive manufacturing techniques for fabricating such architectured ceramics.

In this study, an effective and efficient manufacturing tool offering a near-net shaping material removal system was used to fabricate topologically interlocking ceramics. A picosecond pulsed fiber laser system was used to control the kerf taper (cut angle) and achieve deep, high-quality cuts (i.e., the weak interfaces) with a reduced manufacturing period. Topologically interlocked panels were chosen for this investigation, with several building block sizes and interlocking angles tested. The panels were subjected to low-velocity impact loads to assess the deflection characteristics of the designed architectures. The deformation and toughening mechanisms were further explored to develop an understanding of the structure–property relationship for each of the manufactured panels. While this work focuses on the implementation of topologically interlocked panels, a wide range of architectured geometries can be developed using the delineated methods.

## Methods and materials

### High precision laser system

**Figure 1:**
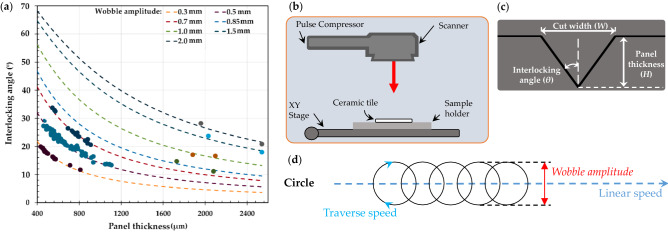
(**a**) Geometric dependence of interlocking angle to the panel thickness, (**b**) the laser system and its equipment, (**c**) schematic of developed cut profiles, and (**d**) circular wobble pattern shapes and its parameters.

The manufacturing of topologically interlocked ceramic panels is a complex process requiring fundamental understanding of the optical properties of the substrate (i.e., alumina). One method previously developed^22,23^, used a circular wobble pattern controlled by the laser scanner to minimized the build-up of residual heat, the formation of microcracks, and material property changes. An Ytterbium picosecond fiber laser (YLPP-25-3-50-R, IPG Photonics, USA) was used for the laser machining process (see Fig. 1a,b). A parametric study was conducted to assess the quality and precision of cuts to ensure that the desired interlocking angles could be effectively manufactured. Control of the kerf taper in the alumina samples was thus achieved via selected scanning parameters. The interlocking angle of ablated cuts with ‘V-shape’ profiles can be determined using $\theta =\mathrm{arctan}\left(\frac{W}{2\mathrm{H}}\right)$, a function of the panel thickness (*H*) and wobble amplitude (*W*). Data gathered from various ablation experiments is plotted against the geometric approximation, as shown in Fig. 1a. The plotted experimental data shows a strong correlation with the theoretical formulation for the tested wobble amplitudes, illustrating methodology for the kerf taper and interlocking angle control. For example, a through cut using a 0.3–2.0 mm range of wobble amplitudes could theoretically produce interlocking angles ranging from 3.4° to 21.5°. However, there is a practical limit that exists for the wobble amplitudes less than 1.0 mm due to clipping of the incident laser spots by the tapered surfaces of the cuts. The clipping phenomenon places part of the laser spot out of focus during the cut, thereby decreasing the energy density of the cut area. With decreasing wobble amplitudes, clipping is more significant, which hinders the laser’s ability to ablate material within the cut region and perform through-cuts on 2.54 mm thick ceramic panels. Therefore, the long processing times resulting from the decreased efficiency does not result in the predicted interlocking angle by the wobble amplitudes of 0.5 mm or less viable for cut depths greater than 1.2 mm. Finally, as the amplitude of the circular wobble increases, the beam clipping decreases due to the wider cut geometry. Therefore, deeper cuts can only be achieved for the larger wobble amplitudes with the 220 passes that were required to cut through the 2.54 mm thick ceramic tile (using wobble amplitude of 1.5 mm).

Proposed in Fig. 1a–d is an approach to predict a high-quality architecture, based on wobble amplitudes, wobble frequency, linear traverse speed, and number of passes. Firstly, by inputting an interlocking angle (*θ*) and thickness of the ceramic tile (*H*), a set of practically feasible wobble amplitudes can be calculated based on $\theta =\mathrm{arctan}\left(\frac{W}{2\mathrm{H}}\right)$, where *W* is the width of the laser cut (or wobble amplitude). To prioritize material removal rate over processing time, a set of low wobble frequencies and corresponding linear speeds is chosen, such that the wobble pitch is less than or equal to 30 µm to minimize ceramic artifacts along the cut line and maintain acceptable cut quality. The specific number of passes is then determined based on the amplitude to obtain the final interlocking angle. Finally, the experimental interlocking angle can be compared with the initial inputs to adjust the wobble amplitude or number of passes.

### Design and manufacturing

**Figure 2:**
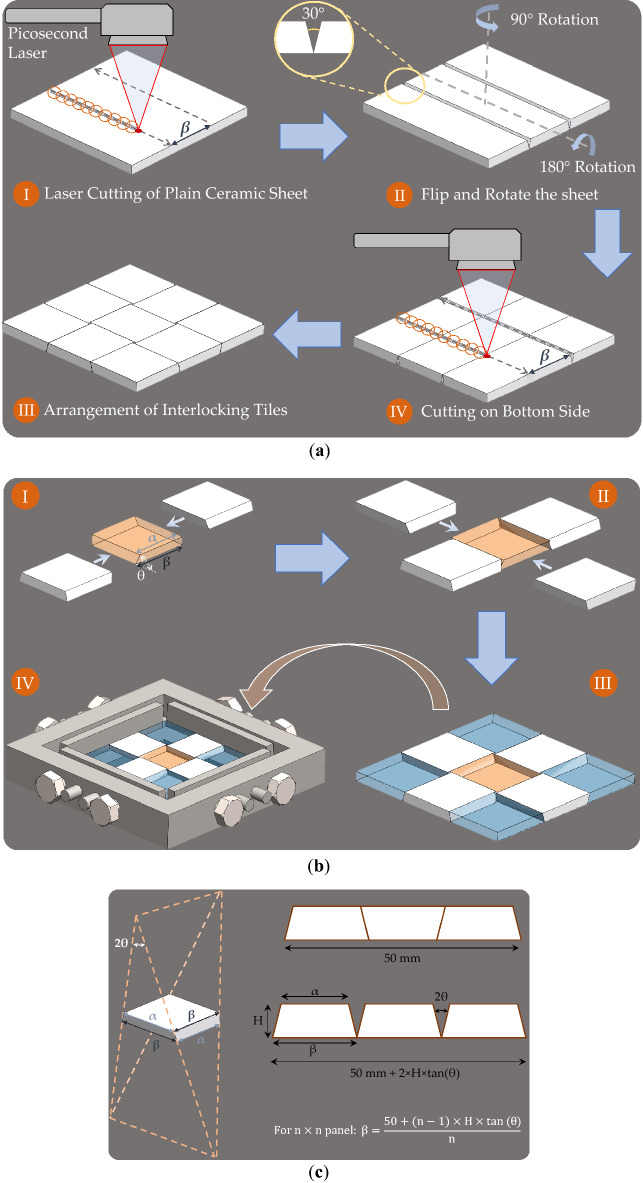
(**a**) Manufacturing of a 15° topologically interlocked ceramics using the laser material removal system. The circular pattern has been exaggerated for visualization; in reality, the diameter corresponds to the cut width. (**b**) Schematic of the assembly set-up for an architectured panel and the fixture. The four profiles with the power screws at the sides of the structure were used to impose an in-plane fixed confinement required for interlocking, and (**c**) line spacing to achieve a 50 × 50 mm^2^ interlocking panel.

Alumina ceramic panels (dimensions = 113.24 × 113.24 × 2.54 mm^3^, high-tolerance fired non-porous alumina ceramic with 96% material composition and a density of 3875 kg/m^3^, McMaster-Carr) were cut into smaller square panels using a diamond saw (M0D31, Struers, Denmark). The square panels were cut such that the final dimensions of the final topologically interlocked panels were 50 × 50 mm^2^. The data in Table 1 conveys the sizing of the square panels based on the target kerf taper or interlocking angle. In addition, the dependency of the kerf taper to the wobble amplitude of the circular laser pattern is presented. Figure 2 illustrates the manufacturing steps to manufacture the topologically interlocked alumina panel using the picosecond laser. Two lines, spaced by parameter *β*, are cut through the alumina samples (i.e., > 2.54 mm). For example, a final interlocking angle of 15° is targeted and the corresponding wobble amplitude of 1.337 mm is set. This produced a measured cut angle of 30° of the through cut sample. The square sample is then rotated 90° about the vertical axis and flipped on the bottom side to machine two additional cuts through the sample. The final interlocking panel is assembled by rotating the edge tiles as shown in Fig. 2b. The sample was then taped and transferred to a steel fixture equipped with power bolts which were adjusted to confine the panels with no pre-compression applied. Once the building blocks were placed in the fixture, the tape was removed.

**Table 1:** Overview of the achievable interlocking angles with the corresponding wobble amplitudes and square panel dimensions.

Interlocking angle	Wobble amplitude (mm)	3 × 3 panel			5 × 5 panel			7 × 7 panel		
		Cutline spacing, *β* (mm)	Dimension of uncut panel (mm)	Lateral size of the blocks (mm)	Cutline spacing, *β* (mm)	Dimension of uncut panel (mm)	Lateral size of the blocks (mm)	Cutline spacing, *β* (mm)	Dimension of uncut panel (mm)	Lateral size of the blocks (mm)
20**°**	1.80	17.28	51.85	1.67	10.74	53.69	1.00	7.94	55.55	0.71
25**°**	2.35	17.46	52.37		10.95	54.74		8.16	57.11	
30**°**	2.90	18.64	52.93		11.17	55.87		8.40	58.79

The developed laser processing scheme has been based on previous work which involves the use of circular wobble pattern to achieve precision deep cuts. Ablation for depths up to 2.54 mm requires the adjustment of parameters during the laser process, primary increases in laser energy density and focal position. Shallow angle cuts (e.g. 20° or lower) require greater processing times due to the lower material removal rates and consecutive parameter adjustment compared to the wider angled cuts (e.g. 25° and 30°). The scheme implemented for the specific picosecond laser system used is shown in Table 2. The energy density of the ablated cuts was modified by decreasing the wobble frequency and speed to achieve adequate material removal at greater depths. For shallower angles, these were adjusted several times to achieve through-cuts. The focal position was modified after 300 passes to refocus the Gaussian beam at the cut geometry on the alumina surface. For the wider angles, only one adjustment step was necessary to achieve through cuts. Consequently, the processing time to manufacture each panel is dependent on the interlocking angle and number of tiles. Panels using the 3 × 3, 5 × 5, and 7 × 7 interlocking tiles use different line lengths and line spacings to ensure that the final interlocking panel dimension is a consistent 50 × 50 mm^2^. These dimensions are calculated using the geometric relationships shown in Fig. 2c. This figure also represents the two distinct tapers on the ceramic tile which are manufactured to ensure that the edges mesh with adjacent tiles. The value for *β* corresponds to the line spacing of the cuts by the picosecond laser and varies depending on the number of tiles and interlocking angle. Prior to manufacturing the panels, a scheme is designed in the picosecond laser’s software to control the spacing of the cut lines and the dimension of the panel using the calculated *β* value. In this study, the architectures (i.e., interlocking angle and block size) of the panels were changed, but their overall dimensions were kept constant. In addition to the architectured ceramics, monolithic ones with identical overall dimensions and thickness (i.e., 2.54 mm) were manufactured and tested for a comparison purpose. Cut panels are assembled using a steel fixture with four adjustable clamps. Figure 2b demonstrates the 3 × 3 panel assembly on the steel fixture which no compressive forces are applied in the planar directions. The interlocked panels are then tested under the impact loading.

**Table 2:** Picosecond laser scheme to achieve deep cuts on alumina.

Interlocking angle	Number of passes	Wobble frequency (Hz)	Speed (mm/s)	Focal position (mm)
20°	0–80	300	8	94.0
	81–300	200	6	94.0
	301–560	150	4	94.5
	561–600	100	3	94.5
25° and 30**°**	0–450	300	8	94.0
	451–600	150	4	94.5

### Experimental test configurations

The architectured ceramics were impacted using a low-velocity drop weight machine based on the guidelines given in the ASTM standard D3763^24^. The 5-mm-hemispherical impactor having a mass of 1030 g was used to apply the load to the structure. The hemisphere was positioned in the middle of the structure. The initial impact velocity was set at 1.70 m/s. The impactor load and impactor velocity throughout the test were monitored and recorded using a load cell (penetration piezoelectric load sensor, with a load capacity of 22.5 kN) and velocity detector (a photo detector block and a flag), respectively.

### Digital image correlation

**Figure 3:**
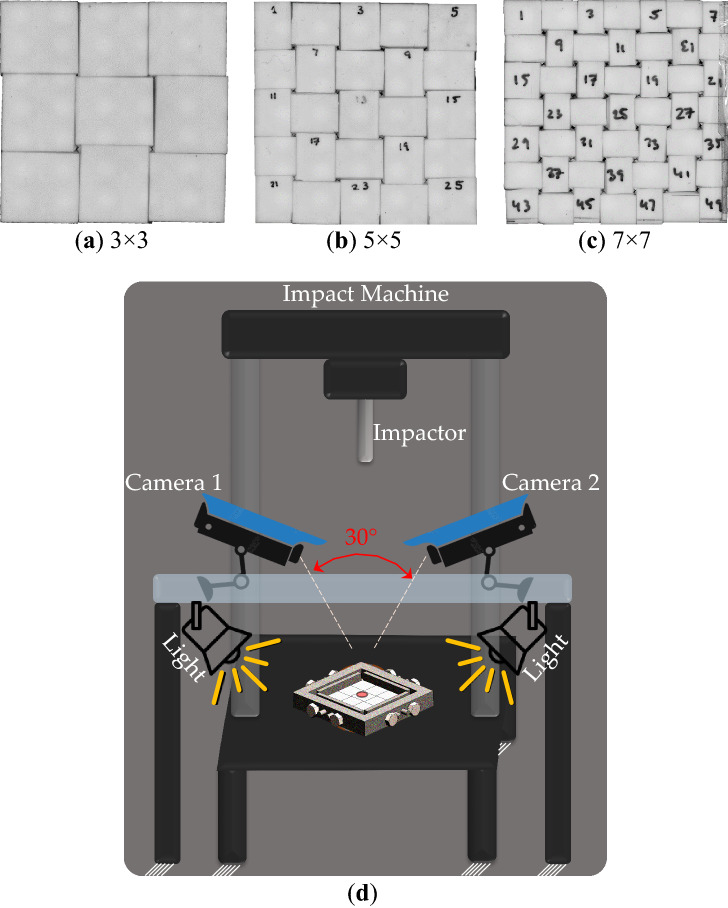
Topologically interlocked ceramics: (**a**) 3 × 3, (**b**) 5 × 5, (**c**) 7 × 7 arrays of blocks (30°), and (**d**) low-velocity impact test and DIC configurations.

A 3D digital image correlation (DIC) system was used during the impact tests to measure the out-of-plane displacement of the ceramic panels as shown in Fig. 3. The stereoscopic DIC system consisted of two high-speed cameras (Photron SA-X) with two Sigma lenses (28–135 mm f/3.8–5.6 Aspherical IF Macro) set to capture images at 12,500 frames per second (fps) with a resolution of 1024 × 1024 pixels. Two high intensity LED lights (JAB Bullet) were used to provide even illumination. A stochastic speckle pattern was applied to the top surface of the ceramics using a thin tipped black marker^25^. DIC data was analyzed using the Vic-3D software (version 9, Correlated Solutions Inc.).

The DIC algorithm requires two parameters to calculate a displacement field across the area of interest (AOI): subset size and step size. The subset size defines the size of the square regions that the AOI is subdivided into and the step size dictates the data density of the calculated displacement field. For these tests, the subset size had to be chosen carefully for each panel to ensure that data for each ceramic tile was not mistakenly influenced by its neighbouring tiles. The subset and step sizes used for these tests in pixels were (15, 3), (31, 5), and (29, 8) for the 3 × 3-, 5 × 5-, and 7 × 7-block panels, respectively.

This DIC analysis resulted in spatially and temporally dense full field displacement maps. To examine the deflection across the panels over time, a line profile was drawn across the centers of each panel and the out-of-plane displacement across that line profile was extracted at three key stages for each panel: before impact, at max deflection, and at the end of the test. Data was also extracted in the intervals between these stages, providing a total of five line extractions per panel.

## Results

**Figure 4:**
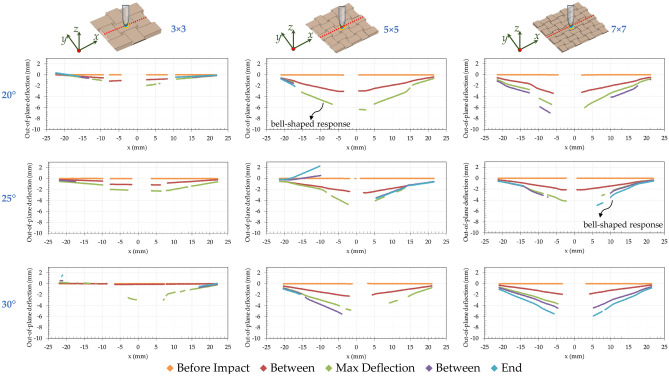
The deflection across the panels obtained by DIC at five stages of loading: architectured ceramics with the interlocking angles (*θ* = 20°, 25°, and 30°) and number of blocks (3 × 3, 5 × 5, and 7 × 7).

When an architectured panel is subjected to an impact load, the relative sliding and rotation of the individual blocks lead to overall large deformations, while they do not deform substantially. Since these relative motions cause frictional sliding, the architectured panel also absorbs much more mechanical energy than a plain panel made with the same material. For plain ceramic; however, much of the energy is dissipated through fracture of brittle materials. The architectures and controlled deformations at the weak interfaces of the blocks leading to ceramics, a brittle material, with large deformation and high energy absorption capabilities. Figure 4 presents the panels’ deflection at five stages of loading (before impact, between, maximum deflection, between, end of the test) along *x* direction and their centers of the topologically interlocked ceramics (interlocking angle of *θ* = 20°, 25°, 30°, and 3 × 3, 5 × 5, 7 × 7 number of blocks). It should be noted that the maximum deflection is recorded before the force dropped. The architectured ceramic panels illustrates significant differences. Particularly, the 5 × 5-block panel has the highest deflection among all the topologically interlocked panels. In addition, the architectured ceramics (such as the 5 × 5-block panel with *θ* = 20° and the 7 × 7-block panel with *θ* = 25°) show a bell-shaped response linked with a progressive failure and indication of tough structures. The architectured panels failed by a progressive push-out of the center block by the impactor. The effects of the interlocking angle on impact properties of the architectured ceramics are observed in Fig. 4. The panels with the lower interlocking angles have a higher deflection. When comparing number of blocks, the ceramic panels with the 5 × 5 arrays of blocks have a 310% higher deflection, compared to the panels with the 3 × 3 arrays of blocks with the same interlocking angle (see Fig. 4).

**Figure 5:**
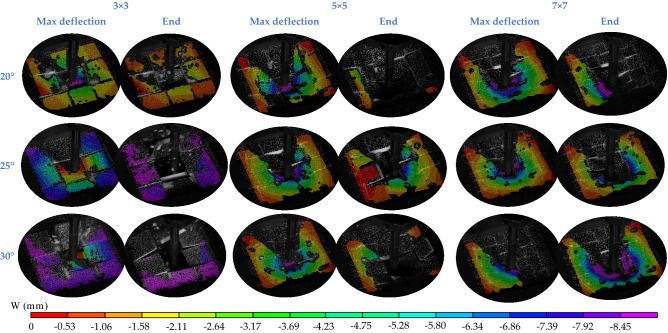
Impact testing of ceramic panels: the 3D DIC displacement field in *z* direction for the interlocked ceramics with the interlocking angles (*θ* = 20°, 25°, and 30°) and the number of blocks (3 × 3, 5 × 5, and 7 × 7).

Figure 5 presents the 3D DIC displacement field in the *z* direction for the interlocked ceramics. The architectured panels demonstrate a bell-shaped response and a progressive failure. The architectured panels failed by a progressive push-out of the center tile by the impactor, while the rest of the tiles remain intact. No flexural cracks have been observed in the architectured panels. Segmenting the panels into building blocks decreases the span over which the flexural stresses are developed leading to an increase in the force required to induce flexural cracks. Since the force required to develop flexural cracks increases, no flexural cracks can be observed in the architectured panels. The architectured ceramic panel is made out of building blocks and its span reduces (i.e., 3 × 3-, 5 × 5-, and 7 × 7-block panel = 1.67, 1, and 0.71 mm, respectively).

**Figure 6:**
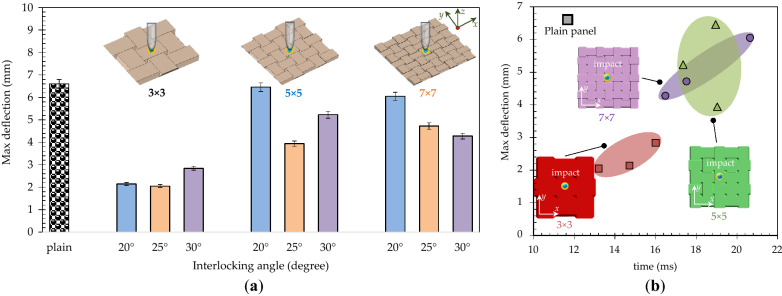
The properties of the architectured and plain ceramics: (**a**) maximum deformation, and (**b**) maximum deformation versus time at maximum deformation of the panels as functions of interlocking angle (*θ* = 20°, 25°, and 30°) and number of blocks (3 × 3, 5 × 5, and 7 × 7).

Figure 6a,b present the maximum deformation and maximum deformation versus time when the maximum deformation occurs for the architectured and plain ceramic as functions of the interlocking angle (*θ* = 20°, 25°, and 30°) and the number of blocks (3 × 3, 5 × 5, and 7 × 7). The observed differences are translated into variations in stiffness, strength and energy absorption. The plain panel deforms the same as much as the 5 × 5-block panel with *θ* = 20° does. Generally, the panels with a smaller interlocking angle showed a higher maximum deformation except for the 3 × 3- block panel. It is seen from Fig. 6b that the 5 × 5- and 7 × 7- block panels took more time to reach to their maximum deflections compared to the 3 × 3- block panel. The 5 × 5- and 7 × 7- block panels also show a higher maximum deflection (see Fig. 6a). The strengths of the architectured ceramics are expected to notably be less than that of the plain panel due to frictional interfaces in the material system. However, this is advantageous for flexible protection such as in personal protective equipment. The improvement in energy absorption provides the ceramic system with multi-hit resistant. Importantly, the architectured ceramics can have less of the response load transferred to the system, which is another advantage for armor and shielding applications. The stiffness of the panels is expected to increase with the interlocking angle because of increased mechanical interaction between the blocks.

## Conclusion

We demonstrated a highly efficient and versatile subtractive manufacturing technique using a picosecond fiber laser system. By optimizing critical laser process parameters in the material removal system, fabrication and concept of deformation properties enhancement by topologically interlocking ceramics were presented with a potential scalability. The nature of the developed laser system allows for controlling the architectures with a high degree of precision. This precise control on the multi-scale level of the architectured ceramics makes our technique uniquely advantageous in the potential scale-up advanced ceramic manufacturing. The robustness of the technique further allows hybrid fabrication of engineered structures of drastically different materials. It is seen that the  interlocked panel has a tendency to laterally expand under the impact loading due to the tapered design of building blocks. Since the lateral expansion is limited by the fixture, in-plane compression is exhibited within the ceramic. Part of the in-plane compression (or elastic energy) is maintained within the panel after test completion because the building blocks are pressed into each other. The deflection decreases with the interlocking angle and increases with increasing the number of blocks. By increasing the number of building blocks of an architectured panel, the manufacturability becomes more time consuming and challenging. However, an architectured panel with a higher number of building blocks (which is less stiff) is more suitable for the applications where comfort plays a more important role (i.e., protective personnel equipment). On the contrary, the architectured panels with the less number of building blocks have higher strength, which is an advantage for armor and shielding applications. The developed design strategy demonstrates a novel design space for the architectured ceramics covering a broad range of engineering applications by tuning the interlocking angle, building block size, and varying the size and angle over the panel width. The developed ceramic panels can be used for armor applications (i.e., personnel protective equipment)^26^ or for thermal barrier systems^27^.

## Data Availability

The datasets generated during and/or analysed during the current study are available from the corresponding author on reasonable request.
